# Water Pipeline Leakage Detection Based on Machine Learning and Wireless Sensor Networks

**DOI:** 10.3390/s19235086

**Published:** 2019-11-21

**Authors:** Yang Liu, Xuehui Ma, Yuting Li, Yong Tie, Yinghui Zhang, Jing Gao

**Affiliations:** 1 College of Electronic Information Engineering, Inner Mongolia University, Hohhot 010021, China; yangliu@imu.edu.cn (Y.L.); xuehuimaimu@163.com (X.M.); yutingli@mail.imu.edu.cn (Y.L.); zhangyinghui_imu@163.com (Y.Z.); 2 Department of Electrical and Computer Engineering, University of Nebraska-Lincoln, Lincoln, NE 68588, USA; 3 Tianjin Key Laboratory of Wireless Mobile Communications and Power Transmission, Tianjin Normal University, Tianjin 300387, China; jing401@126.com

**Keywords:** leakage detection, wireless sensor networks, machine learning, leakage triggered networking

## Abstract

The detection of water pipeline leakage is important to ensure that water supply networks can operate safely and conserve water resources. To address the lack of intelligent and the low efficiency of conventional leakage detection methods, this paper designs a leakage detection method based on machine learning and wireless sensor networks (WSNs). The system employs wireless sensors installed on pipelines to collect data and utilizes the 4G network to perform remote data transmission. A leakage triggered networking method is proposed to reduce the wireless sensor network’s energy consumption and prolong the system life cycle effectively. To enhance the precision and intelligence of leakage detection, we propose a leakage identification method that employs the intrinsic mode function, approximate entropy, and principal component analysis to construct a signal feature set and that uses a support vector machine (SVM) as a classifier to perform leakage detection. Simulation analysis and experimental results indicate that the proposed leakage identification method can effectively identify the water pipeline leakage and has lower energy consumption than the networking methods used in conventional wireless sensor networks.

## 1. Introduction

Water provides a material basis for human life and the survival of all living things, and it is an indispensable natural resource needed for the development of human society. Due to population growth, economic development, and changing consumption patterns, the demand for water resources is increasing rapidly, and this increasing demand is expected to accelerate greatly during the next 20 years [1]. However, the wastage of water resources caused by leakage from water pipelines is an important problem [2]. A study conducted by the World Bank indicated that the leakage from water pipeline exceeds 48.6 billion $m^{3}$ annually, and the corresponding annual economic losses are approximately US$14.6 billion [3]. According to the 2012 Green City Index statistics, water pipeline leakage rates exceed 10% in one third of countries worldwide. For example, the average water pipeline leakage rate is 23% in the EU, 13% in the U.S. and Canada, 22% in Asia, 35% in Latin America, and 30% in Africa [4,5]. Accordingly, research on high performance water pipeline leakage detection technologies has great significance for the protection of water resources and the promotion of economic development.

The main causes of water pipeline leakage include the corrosive nature of the soil, deficient pipe material quality, the temperature and pressure, failure to employ standard pipe laying methods, geological changes, and human damage [6,7,8]. For instance, deterioration caused by the pressure of the soil may lead to uneven pipeline stress bearing, which can result in leakage from ruptures and breakage. Leakage may also result from damage to pipe junctions caused by external impacts or ruptures caused by the failure to weld junctions during construction. Nevertheless, because the vast majority of water pipelines are located deep underground, leakage is typically not promptly discovered, and when the amount of leakage is large, it is usually not discovered until water begins flowing from the ground surface. Furthermore, since most leaks are relatively small or undetectable, leakage causes substantial waste of water resources [9,10]. Therefore, it is extremely important to detect leakage from underground water pipelines effectively and accurately.

Academic researchers and the industry have conducted major research campaigns and developed numerous effective detection methods. One of the earliest detection methods is the listening system in which detection personnel listen to changes in the volume and sound quality of leakage noise coming from equipment and locate areas of leakage based on these observations [11,12]. This method not only depends on the experience of the detection personnel, but is highly labor intensive and unreliable due to the large areas of water pipeline networks. Ground penetrating radar can determine the locations of pipeline leaks through the detection of soil voids caused by water leakage. However, because of the complex differences in the geological structure in different areas, this method has poor applicability and is very expensive [13,14]. Other studies have presented leakage detection inspired by the changes in the internal pressure of a water pipeline, such as the pressure gradient method, negative pressure wave method, and flow rate balance method [15,16,17]. Although these methods are relatively sensitive to a pipeline’s flow rate and pressure, they tend to give false positive results when the flow rate fluctuations are large because the flow rate in water pipeline networks will fluctuate continuously. It has been found that the spectrum of leakage signals is concentrated, and the pipeline vibration frequency is correlated with the leakage state [18]. This characteristic can be employed in conjunction with the spectral analysis of signals from piezoelectric accelerometers to perform leakage detection. However, when the environmental noise has a similar frequency spectrum to that of the leakage signals, this method also has a tendency to give false positives. Another study used the linear predictive coding coefficient (LPCC) of acoustic leakage signals and a hidden Markov model (HMM) to improve the ability to distinguish the leakage signal from interfering environmental signals [19]. However, the detection error of this method tends to increase with the length of time that the system is in use. Furthermore, in [20,21,22], some detection methods based on acoustic signals, such as through hydrophones, have proven that effectively leak detection can be achieved in a longer range of detection in water pipelines. These methods exhibit a high sensitivity to leaks in the range of interest, appearing appropriate for early leak detection. Additionally, the works in [23,24,25] have applied machine learning to pipeline monitoring and have achieved excellent results. Moreover, research on large scale water pipeline network systems has used methods such as real-time modeling to compare measured pipeline network data with flow rate model predictions [26,27,28,29], but problems such as high modeling difficulty and high computational loads in real applications limit the use of these methods. Researchers have achieved impressive results in research in the field of tap water pipeline leakage detection and location. However, the large scale of water pipeline networks and the extreme complexity of the network architectures, environmental conditions, and geological factors still pose significant challenges for the real-time monitoring of water supply pipeline networks and intelligent leakage detection.

Wireless sensor networks (WSNs) provide an efficient way to address these issues. Due to their outstanding sensing ability, communication protocols, processor speed, and data collection advantages, wireless sensor network technologies have been widely applied in the field of monitoring [30,31,32,33,34]. In [35], PipeNet, a system based on wireless sensor networks, was proposed. It aims to monitor water flow and detect leaks by attaching acoustic and vibration sensors to large bulk-water pipelines and pressure sensors to normal pipelines. In contrast to the PipeNet project, the PipeProbe system does not assume that water pipe surfaces are exposed and accessible for sensor module attachment [36]. PipeProbe can be dropped into the source of a water pipeline. During its traversal of the pipeline, it collect the sensor readings necessary for the reconstruction of the 3D spatial layout of the traversed water pipelines. To demonstrate the application and control of a low cost wireless sensor network for a high data rate, WaterWiSe@SG, a wireless sensor network to enable real-time monitoring of a water distribution network in Singapore, was proposed in [37]. The goal of WaterWiSe@SG was to develop generic wireless sensor network capabilities to enable real-time monitoring of a water distribution network.

This paper proposes a water pipeline monitoring system based on wireless sensor networks and a leakage identification method based on support vector machine (SVM). Machine learning can simulate the acquisition of knowledge through human learning activities and can enable the automatic improvement of system performance. As a result, it is widely applied in speech and biological affect identification [38], physiological signal detection [39], body movement identification [40], signal feature detection and identification, etc. [41,42,43,44]. The proposed system employs ZigBee nodes serving as signal collection nodes and uses the 4G network to transmit the signals adopted by the sensors to the data processing center for processing. To address the high networking power consumption that affects conventional wireless sensor networks, we also propose a leakage triggered networking method able to network and perform data transmission from wireless sensor nodes in the vicinity of leakage points, which effectively reduces the network energy consumption and extends its life cycle. Based on the differences in the time-frequency features of leakage and non-leakage signals, we propose a leakage detection method that constructs feature matrices by employing the intrinsic mode function, approximate entropy, and principal component analysis (PCA) and that uses SVM as a classifier to identify the leaks. Our experiment is performed along an exposed aluminum-plastic composite pipe, with a diameter of 27 mm. The CT1010 acceleration sensor is used in this experiment due to its sensitivity. During our experiments, the water pressure is no less than 0.3 MPa. The detectable leaking flow rate is calculated to be approximately 2.5 cm${}^{3}$/s based on the pipe pressure. Experimental and simulation results demonstrate that the proposed methods can effectively detect the leakage and prolong the lifetime of the wireless sensor network.

The rest of the paper is organized as follows. The implementation of the leakage monitoring system is introduced in Section 2. In Section 3, the leakage triggered networking solution for wireless sensor networks that are able to network sensors receiving leakage signals is developed. We propose a leakage detection method based on time-frequency features and SVM in Section 4. Experimental and simulations results are reported in Section 5. Conclusions are presented in Section 6.

## 2. Water Pipeline Leakage Monitoring System Based on ZigBee Technology

**Figure 1:**
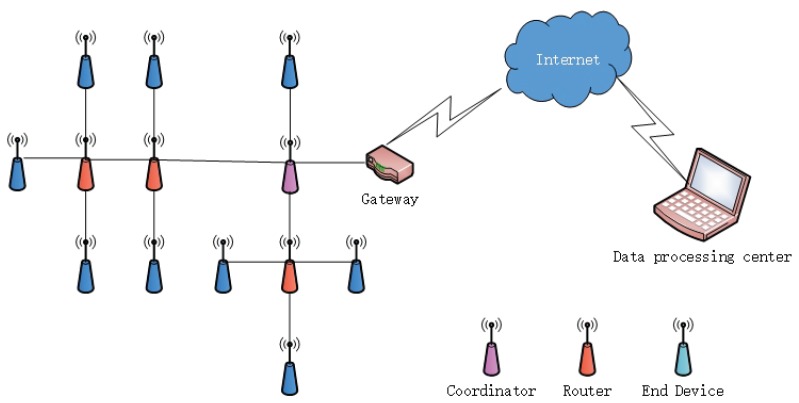
System architecture for pipeline monitoring.

We designed a water pipeline monitoring system based on a ZigBee and 4G wireless communication system. It included a ZigBee wireless network, a gateway, and a data processing center. The system architecture is shown in Figure 1. The ZigBee wireless sensor was constructed from terminal nodes, coordinators, and routers. The terminal node employed an acoustic sensor to acquire signals from pipeline leakage, and the coordinator relied on serial ports and a gateway to establish a linkage and maintain the two-way transmission of data and control commands between the terminal nodes and the gateway. The gateway also took advantage of the 4G network to upload data collected by the sensors to the host for storage and processing.

**Figure 2:**
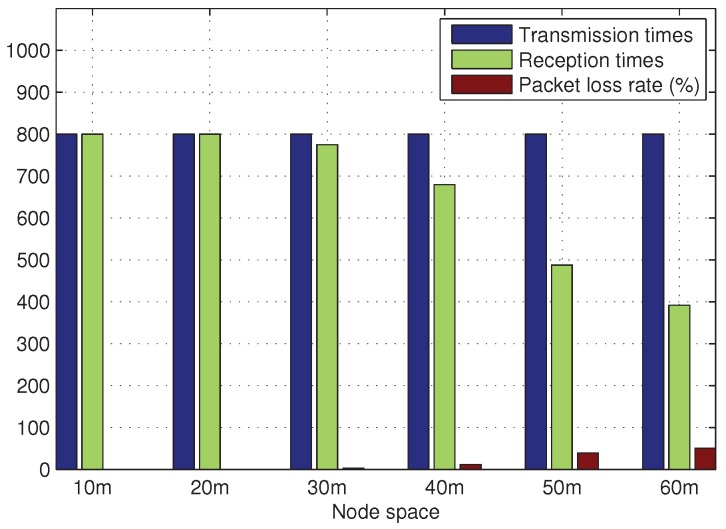
CC2530 packet loss rate under NLOS conditions.

In this system, the ZigBee nodes employed CC2530, and the Zstack protocol was used for data transmission. The system’s gateway employed STM32F103VET6 as its chief control element and used a built-in 4G module to perform networking functions. The gateway and ZigBee coordinator communicated via a serial port, which enabled the data processing center to monitor and control the ZigBee wireless sensor network. The gateway used its built-in multithreading TCP server to monitor requests from the host and transmit data after establishing a connection. The host’s functions included display of the remote pipeline monitoring system, interactive controls, and signal processing. The software on the host was developed using C# and SQL, and the TCP protocol and TCP server on the gateway established network connections and received control commands and data transmissions from the ZigBee wireless sensor network. The CC2530 has a nominal maximum operating distance of 75 m, and research has indicated that ZigBee nodes have a reliable operating range of 60 m in a line-of-sight (LOS) environment [45,46]. The data acquisition card used in this paper was NI’s MCC BTH-1208LS, which was used to save data to the host computer for analyzing signals. CT1010 acceleration sensor was used in this experiment due to its sensitivity, which was matched with data acquisition card. Figure 2 shows the results of testing the packet loss rate of the system’s terminal nodes in a non-line-of-sight (NLOS) environment as the interval between the nodes changes. To ensure the reliability of the system’s data transmissions, the intervals between the CC2530 nodes should be kept at approximately 30 m.

## 3. Leakage Triggered ZigBee Networking

In actual water pipeline monitoring environments, a large number of terminal sensors needs to be installed along pipelines. Since the pipeline leakage is a low probability event, all of the sensors working at the same time will result in a significant waste of energy. Furthermore, leakage in water supply pipelines occurs randomly, and pipeline systems must be monitored in real time. To reduce energy consumption and increase network lifetime, we propose a leakage triggered networking method.

**Figure 3:**
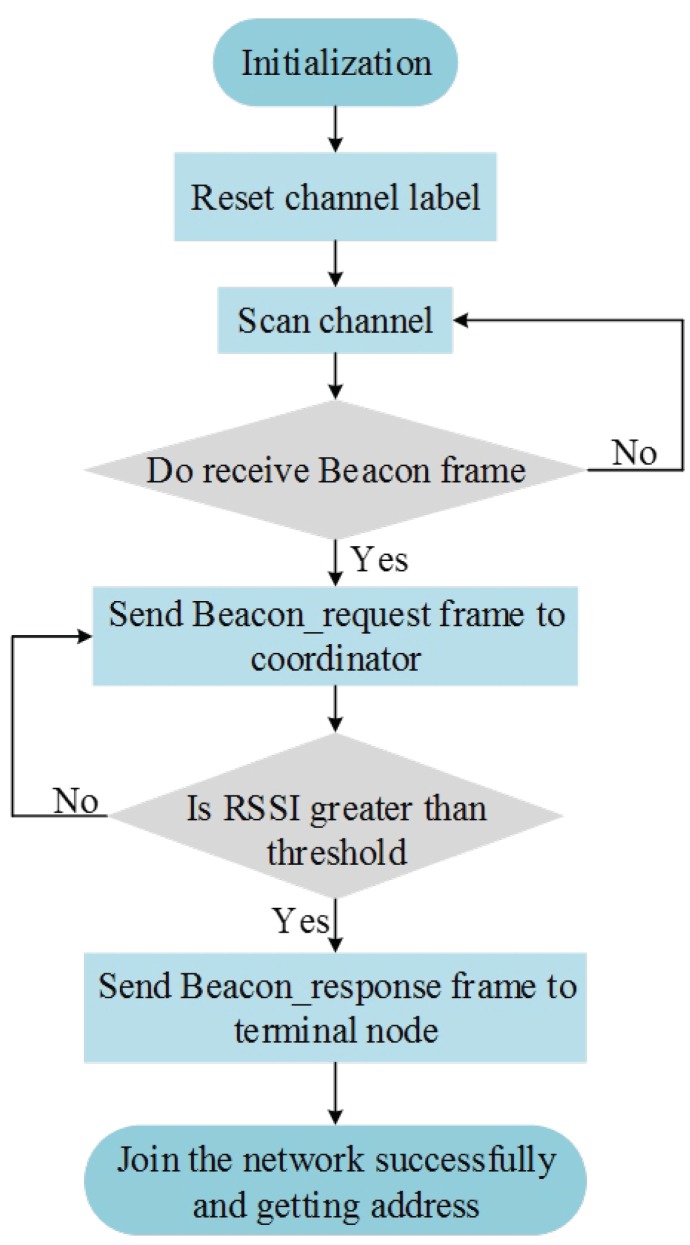
Flowchart of the nodes joining the network.

The ZigBee networking method includes initialization networking and triggered networking. The topological structure of the network provides an important basis for ZigBee networking; in view of the structural characteristics and distribution of water supply pipelines, this paper employed a network topology. To achieve initialization networking, the first step was to determine the coordinator nodes and set their signal channels and network ID numbers, which would initialize the network. Non-coordinator nodes were then added to the network. Figure 3 mainly interprets how the nodes join the network. To ensure that the number of terminal nodes installed at each relay node (i.e., router node) was balanced, it would add received signal strength indicator (RSSI) information to each Beacon_request frame when each terminal node in this solution sends a network join request. In accordance with the RSSI values, routing nodes provided joining service to the terminal nodes. To collect leakage signals effectively and reliably, it is necessary to set reasonable threshold values for the RSSI. If the RSSI values of the terminal nodes are smaller than the threshold, the routing nodes will not process the request of terminal nodes. If the RSSI values are greater than the threshold, the routing nodes will record the terminal nodes’ information and ensure that they can join the network.

After the network was initialized, to reduce the network power consumption and prolong the network lifetime, this paper designed three types of control frames (i.e., join frame, active frame, and wave frame), which can be used to trigger the network according to the leakage detection results. The structures of the join frame, active frame, and wave frame are shown in Table 1, Table 2 and Table 3. The Sou_address field is the original address, which is the routing node address. The nodes rely on this field to determine whether they have been activated and are working. The Des_address field is the destination address and constitutes the address of a routing node that has joined the network. The PANID is the network address. In the join frames, the join_result field is the result of networking; a result of zero indicates that the networking of a routing node has failed, and a result of one indicates that it has been successful. In the active frame, the Act_address field is the active address, and child nodes will join the network after receiving this information and then perform data sampling. In the wave frames, the Position field records the actual physical address information. Because this paper mainly considered the networking solution and its performance, the Position field was not used for node location.

**Figure 4:**
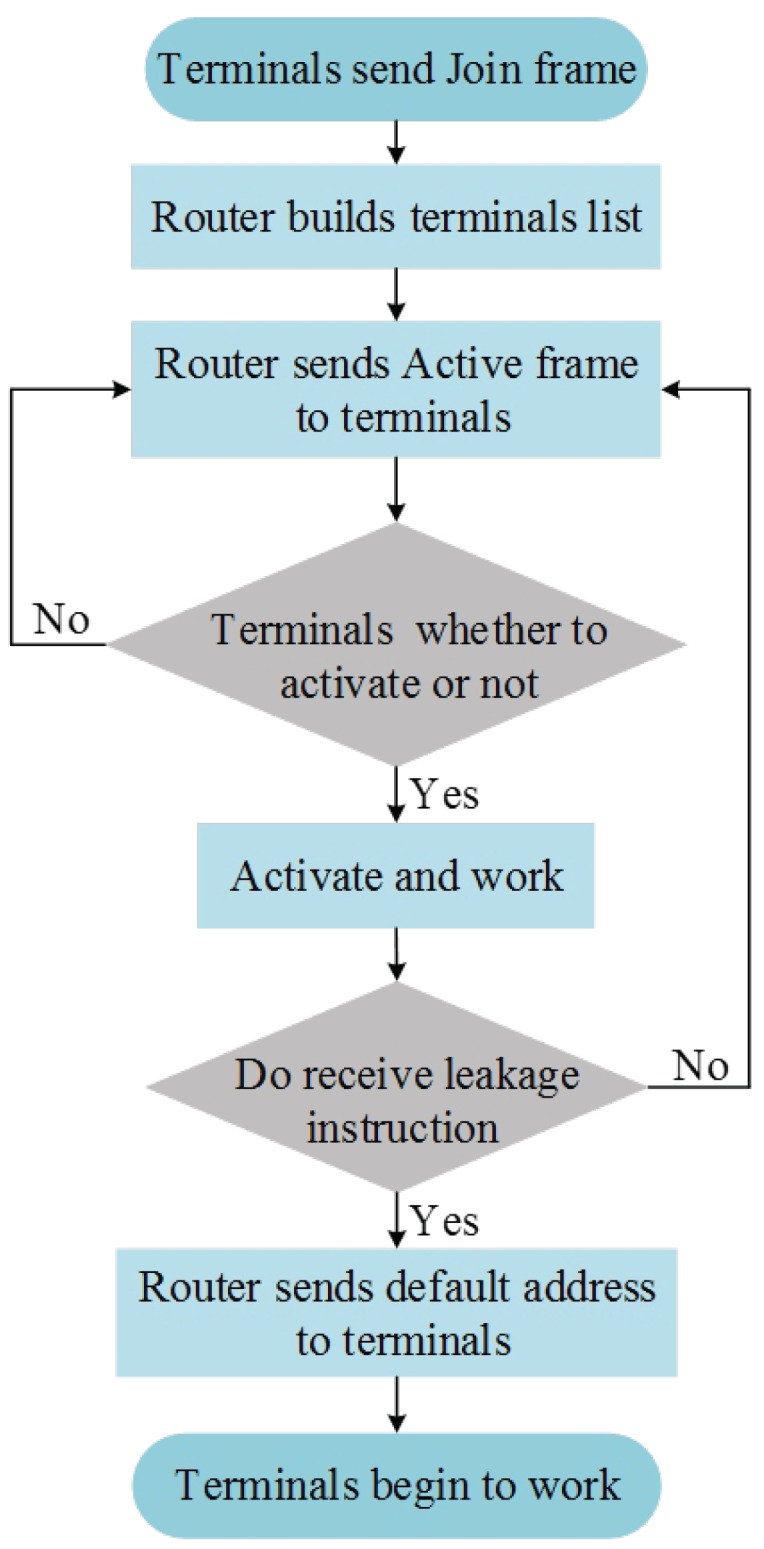
Flowchart of the ZigBee network leakage triggered networking.

Figure 4 shows the leakage triggered networking flowchart. The terminal nodes send Join frames to the routing nodes to compile a list of terminal nodes. After the routing-terminal relationship has been constructed, the routing nodes will send Active frames to the terminal nodes. When receiving Active frames, the terminal nodes will then determine whether they are the destination nodes. If a node is a destination node, it will activate itself and perform data sampling. If a node is not a destination node, it will enter a sleep monitoring state and wait for the next Active frame. Once a node in the working state determines that a signal it has received is a leakage signal, the routing node will send the preset leakage triggered address to the nodes on the terminal nodes list. All of the nodes on the list receiving that address will activate themselves and initiate signal sampling and transmission. This method enables routing nodes to control the working status of the terminal nodes. After the networking is completed, data transmission is performed using the ZigBee routing protocol.

## 4. Leakage Detection by Using Machine Learning and Time-Frequency Features

### 4.1. Time-Frequency Analysis of the Acoustic Leakage Signal

#### 4.1.1. Spectrum Density Feature

Many studies have indicated that the components of the spectra of leakage signals are chiefly concentrated within specific bands. As a result, the differences between signal spectra can be employed as pipeline leakage identification characteristics. To extract the differences of the signal spectral density, we used empirical mode decomposition (EMD) to perform a time-frequency analysis of pipeline signals and proposed a frequency domain feature for detection.

EMD can selectively decompose the signal as the sum of a finite number of intrinsic mode functions (IMFs) [47], enabling multiple IMFs to be used in the multiscale analysis of the spectrum density of leakage signals. An analytic function z(t) can be expressed as:

(1) $$z\left(t\right)=x\left(t\right)+j\hat{x}\left(t\right)=a\left(t\right)e^{j\Phi (t)},$$

where $\hat{x}\left(t\right)$ is the Hilbert transform of a signal x(t) and Φ(t) is given by:

(2) $$\Phi \left(t\right)=\arctan\frac{\hat{x}\left(t\right)}{x(t)}.$$

Finding the derivative of the phase function with respect to time enables the signal analysis instantaneous frequency function to be determined as:

(3) $$f\left(t\right)=\frac{1}{2\pi }\frac{d\Phi (t)}{dt}.$$

The definition of the instantaneous frequency shows that although the instantaneous frequency may take the form of a meaningless negative frequency under certain situations, if the instantaneous frequency is positive throughout a certain period of time, then the x(t) can be termed the IMF. Accordingly, an IMF must satisfy the following two conditions:

(1) The number of extreme points is $N_{e}$ (including the minimum and maximum values), which is the same as or no more than one from the number of zero crossing points $N_{s}$,
  (4) $$\left(N_{s}-1\right)\leq N_{e}\leq \left(N_{s}+1\right).$$
(2) At an arbitrary time $t_{i}$ within the time period, the mean of the upper envelope determined by the local maximum and the lower envelope determined by the local minimum is zero,
  (5) $$\left[x_{max}\left(t\right)+x_{min}\left(t\right)\right]/2=0,\;t_{i}\in \left[t_{a},t_{b}\right].$$

Generally, a signal can include multiple IMFs. The EMD method can be used to extract the IMFs from a signal. To derive the spectrum density features of a leakage signal, we used the EMD method to process the signal, which will yield the IMFs of that signal. All the extreme points of the original signal x(t) are connected with a cubic spline curve, yielding the upper and lower envelopes of x(t), which causes the signal to be contained between the two envelopes. We assumed that the function formed by means of the two envelopes is m(t). Subtracting m(t) from the original signal x(t),

(6) $$h_{1}\left(t\right)=x\left(t\right)-m\left(t\right).$$

We now check whether $h_{1}\left(t\right)$ satisfies the two conditions of the IMF. If it does not satisfy them, repeat (6) until the IMF conditions are satisfied. $h_{1}\left(t\right)$ at this time is expressed as $c_{1}\left(t\right)$, where $c_{1}\left(t\right)$ is the first IMF of the signal x(t),

(7) $$c_{1}\left(t\right)=h_{1}\left(t\right).$$

Next, $c_{1}\left(t\right)$ is subtracted from the original signal x(t) to obtain the new signal $r_{1}\left(t\right)$,

(8) $$r_{1}\left(t\right)=x\left(t\right)-c_{1}\left(t\right).$$

Repeating (6) until the IMF conditions are satisfied, the first IMF of $r_{1}\left(t\right)$ is obtained, which is the second IMF of x(t) and is denoted as $c_{2}\left(t\right)$. Continuing in the same manner, we can progressively derive the $m^{\mathrm{th}}$ IMF $c_{m}\left(t\right)$ of the signal x(t) and the remainder $r_{m}\left(t\right)$.

Using the foregoing steps, the original signal x(t) can be decomposed into the sum of IMFs and a remainder,

(9) $$x\left(t\right)=\sum_{i=1}^{m}c_{i}\left(t\right)+r_{m}\left(t\right).$$

In general, the IMF condition (2) is difficult to satisfy; thus, a stopping criterion is generally established. When the stopping criterion has been satisfied, Condition (2) can be considered to have been met. For this reason, we set the standard deviation between two consecutive processing results as $S_{d}$,

(10) $$S_{d}=\sum_{n=0}^{N-1}\frac{{\left|h_{k-1}\left(n\right)-h_{k}\left(n\right)\right|}^{2}}{h_{k}^{2}\left(n\right)},$$

where *N* is the observed signal length and $h_{k-1}\left(n\right)$ and $h_{k}\left(n\right)$ are two consecutive processing results in the process of the IMF derivative. When the standard deviation $S_{d}$ reaches the preset threshold value, we can assume that Condition (2) has been satisfied. It has been indicated in [47] that the threshold value of the standard deviation $S_{d}$ is typically taken as 0.2–0.3.

After the IMFs of a signal have been obtained, we can further obtain the discrete Fourier transform $C_{i}\left(k\right)$ of the IMF components $c_{i}\left(n\right)$ resulting from EMD decomposition,

(11) $$C_{i}\left(k\right)=\sum_{n=0}^{N-1}c_{i}\left(n\right)e^{-j\frac{2\pi }{N}kn}.$$

Deriving the modulus square of $C_{i}\left(k\right)$ yields the IMF power spectrum of the signal,

(12) $$P_{i}\left(k\right)=\frac{1}{N}{\left|C_{i}\left(k\right)\right|}^{2}.$$

We then obtain the mean of Equation (12),

(13) $${\bar{P}}_{i}=\frac{\sum_{k=0}^{N-1}{\left|C_{i}\left(k\right)\right|}^{2}}{N^{2}}.$$

This paper uses the mean value of the IMF power spectrum as the frequency domain feature of the leakage signal.

**Figure 5:**
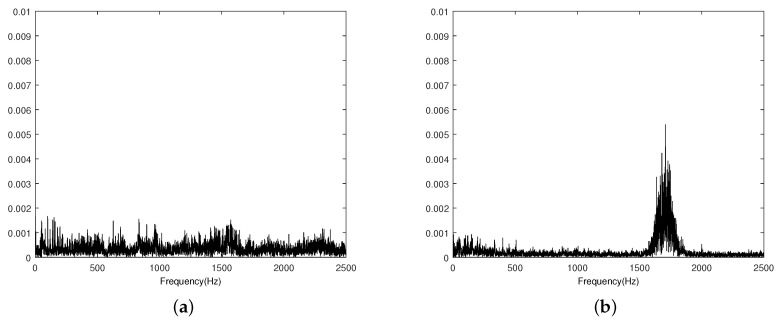
Power spectra of signals from a pipeline. (**a**) Normal signal power spectrum. (**b**) Leakage signal power spectrum.

**Figure 6:**
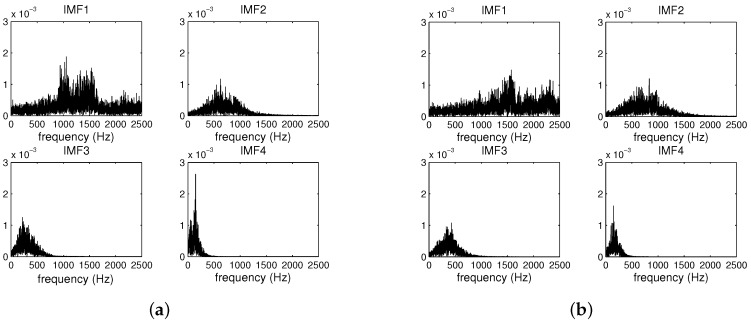
The first four intrinsic mode function (IMF) spectra of the pipeline leakage and non-leakage signal. (**a**) Leakage signal. (**b**) Non-leakage signal.

Figure 5 shows the power spectra of the signals from a pipeline with and without leakage. The results indicate that the frequencies of the acoustic signals leakage were chiefly concentrated near 1.6 kHz, which is consistent with previous research [48]. Figure 6 is the spectra of the first four groups of IMF components obtained by EMD of the pipeline leakage signal and the pipeline non-leakage signal. In this experiment, the threshold value of the standard $S_{d}$ was set as 0.3. Comparing the IMF of each layer of the leakage signal and the non-leakage signal, it can be seen that the main spectrum of the signal was in the first layer IMF. The spectrum of the leakage signal mainly distributed between 1000 Hz and 2000 Hz. The non-leakage signal spectrum was more random and mostly distributed over the entire band.

#### 4.1.2. Signal Complexity Feature

Because pipeline leakage is a localized and low probability event, there should be differences in the time domain composition of leakage signals and non-leakage signals, and the composition of leakage signals should be more complex. Accordingly, the differences of signal composition in time domain can be used to identify leakage signal. The approximate entropy (ApEn) is the conditional probability when similarity is maintained after the dimensions of a similarity vector are increased from *m* to m+1 and is the probability of the new mode when the number of dimensions changes [49]. The greater the probability of the new mode is, the more complex the signal and the greater the corresponding ApEn. Therefore, we used the average ApEn as a time domain feature to quantify signal complexity.

For a sequence u(1), u(2), ⋯, u(N), two sequences with length *m* can be used to construct as x(i)=[u(i),u(i+1),⋯,u(i+m−1)] and x(j)=[u(j),u(j+1),⋯,u(j+m−1)], where i,j≤N−M+1. We then calculate the distance between x(i) and x(j),

(14) $$d\left[x\left(i\right),x\left(j\right)\right]=\max_{k=1,2,\cdots ,m}\left[\left|u(i+m-1)-u(j+k-1)\right|\right].$$

Assuming a threshold value *r*, we determine the number of d[x(i),x(j)]≤r (which is set as *L*) for every i<N−m+1 and calculate the ratio of *L* and the number of vectors,

(15) $$C_{i}^{m}\left(r\right)=\frac{L}{N-m+1}.$$

For all *i* values, we derive the mean $\phi^{m}\left(r\right)$ of $\ln C_{i}^{m}\left(r\right)$,

(16) $$\phi^{m}\left(r\right)=\frac{\sum_{i=1}^{N-m+1}\ln C_{i}^{m}\left(r\right)}{N-m+1}.$$

Increasing *m* by one, we repeat Steps (14)–(16) to obtain $\phi^{m+1}\left(r\right)$, and in accordance with $\phi^{m+1}\left(r\right)$ and $\phi^{m}\left(r\right)$, we can obtain the ApEn value as:

(17) $$\mathrm{ApEn}\left(m,r\right)=\phi^{m}\left(r\right)-\phi^{m+1}\left(r\right).$$

**Figure 7:**
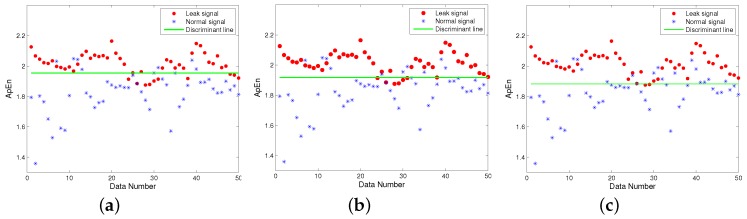
ApEn of signals for different threshold values. (**a**) r=0.3SD. (**b**) r=0.2SD. (**c**) r=0.1SD.

The results showed that the ApEn was a dimensionless scalar quantity, and its value was related to *m* and *r*. To ensure that the ApEn had reasonable statistical characteristics, based on experience, m=2 is usually employed, and *r* was set as 0.1–0.3-times the standard deviation (SD) of the sequence [49]. Figure 7 shows the ApEn of signals before and after leakage for different threshold values. In the experiment, 50 datasets were obtained in each of the two situations, the length of each dataset being 5000, with r=0.3SD, r=0.2SD, r=0.1SD, respectively. The results in Figure 7 show that when r=0.2SD, seven leakage signals were discriminated as normal signals and 10 normal signals were discriminated as leakage signals, with an accuracy of 83%; while in the other two cases, the accuracies were 81% and 80%, respectively. Therefore, it was appropriate to set the threshold value as r=0.2SD. The above analysis indicates that the complexity of the leakage signal should be higher than that of the non-leakage signal, and the complexity can therefore be used to identify leakage.

#### 4.1.3. Signal Principal Component Feature

PCA is a classical feature extraction method that involves the reduction of dimensionality and converts variables into a smaller number of aggregate variables (the principal components). Each principal component is a linear combination of the original variables, and the individual principal components are not mutually correlated. The principal components can convey a vast majority of the information contained in the original variables, and this information is not mutually overlapping. In this paper, we used PCA to analyze the differences between pipeline leakage signals and non-leakage signals.

Assuming that *n* samples are obtained each time from pipeline signals, this can be expressed as $x_{i}={\left(x_{1i},x_{2i},\cdots ,x_{ni}\right)}^{T}$. If we have *m* sets of data $x_{1}$, $x_{2}$, ⋯, $x_{m}$, we can construct an n×m matrix $X=[x_{1}x_{2}\cdots x_{m}]$ as:

(18) $$X=\left[\begin{array}{cccc} x_{11} & x_{12} & \cdots & x_{1m}\\ x_{21} & x_{22} & \cdots & x_{2m}\\ \vdots & \vdots & \ddots & \vdots \\ x_{n1} & x_{n2} & \cdots & x_{nm} \end{array}\right].$$

We used the signal principal components to construct a n×l (0<l≤m) component signal matrix $Y=[y_{1}y_{2}\cdots y_{l}]$, and then constructed a matrix G based on the internal product $g_{ji}=\left[y_{j},x_{i}\right]$ of the principal component signal matrix and original signal matrix,

(19) $$G=Y^{T}\left(X-E\left[X\right]\right).$$

We further chose $g_{j}=\left[g_{j1}g_{j2}\cdots g_{jm}\right],0<j\leq l$ as the feature for the identification of leakage.

### 4.2. Machine Learning Inspired Water Pipeline Leakage Detection

Although the features in Section 4.1 have different characteristics in connection with the identification of pipeline leakage, the use of a single feature for identification is inefficient. For example, if the spectra of leakage and non-leakage signals are significantly different, the mean spectra of the IMFs will have excellent identification ability. However, when there is interference in the same band, this method tends to yield many false results. In addition, when the leakage from a pipeline is relatively small, the mean ApEn will have a poor ability to differentiate between leakage and non-leakage signals.

To increase the accuracy of leakage detection, this paper took advantage of the time-frequency features to construct identification feature sets and used SVM to classify the signal features and thereby determine pipeline leakage. The SVM is an advantageous means of solving small sample problems, nonlinear problems, and problems involving high-dimensional data, e.g., data forecasting, data fitting, and model identification. Assume that $\left(x_{i},y_{i}\right)$ constitutes a training set data sample, where 1≤i≤N, $x_{i}\in R^{d}$ for each sample, *d* is the dimensionality of the input space, and $y_{i}\in \left\{-1,1\right\}$ is the classification label. The training set can be linearly delimited by a hyper-plane that can be expressed as w·x+b=0, where w and *b* are locations that determine the hyper plane. A sample satisfying the following conditions is termed a support vector:

(20) $$y_{i}\left(w\cdot x_{i}+b\right)=1.$$

In fact, the optimal classification of a sample is the solution for the optimal classification hyper plane,

(21) $$\left\{\begin{array}{c} min\phi \left(w,\xi \right)=\frac{1}{2}{∥w∥}^{2}+c\sum_{i=1}^{N}\xi_{i}\\ \;s.t.\;y_{i}\left(w\cdot x_{i}+b\right)\geq 1-\xi_{i} \end{array},\right.$$

where w is the coefficient vector of the classification hyper-plane in the feature space, *b* is the threshold value of the classification plane, $\xi_{i}\left(\xi_{i}\geq 0\right)$ is a relaxation factor included to account for the classification error, and *C* is a penalty factor for the misclassified sample. The optimal classification hyper-plane obtained can be expressed as:

(22) $$w_{o}\cdot x+b_{o}=0.$$

In nonlinear separable situations, a projection function (termed the kernel function) is used to project an input space $R^{d}$ with low dimensionality into a feature space *H* with high dimensionality, which converts the training sample from a linear inseparable problem with low dimensionality into a higher dimensional linear separable problem. At this time, the optimized dual problem is:

(23) $$\left\{\begin{array}{c} \max_{\alpha }\sum_{i=1}^{N}\alpha_{i}-\frac{1}{2}\sum_{i=1}^{N}\alpha_{i}\alpha_{j}y_{i}y_{j}K\left(x_{i},x_{j}\right)\\ \;s.t.\;\sum_{i=1}^{N}y_{i}\alpha_{i}=0,\;0\leq \alpha_{i}\leq C \end{array},\right.$$

where $K\left(x_{i},x_{j}\right)=\Phi \left(x_{i}\right)\cdot \Phi \left(x_{j}\right)$ is the kernel function. Equation (23) shows that an SVM model with a suitable kernel function K(·) must be chosen in the case of a nonlinear separable problem. The decision function corresponding to the use of Equation (23) is:

(24) $$f\left(x\right)=sgn[\sum_{i=1}^{N}\alpha_{i}y_{i}K\left(x_{i},x\right)+b].$$

**Figure 8:**
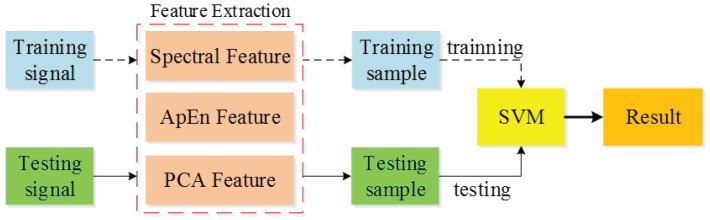
Schematic diagram of the SVM identification model training and optimization.

To improve the accuracy of leakage detection, we must use the training sample and testing sample to optimize the SVM, and the optimization processes are shown in Figure 8. Due to the effects of environmental factors on underground water pipelines, it is necessary to perform signal sampling during different times and at different places to compile sample sets including leakage signals and non-leakage signals. At first, the feature set of the training sample is used to perform SVM training, which creates a preliminary identification model. The feature set of the testing sample is then used to test the trained SVM model. The SVM model is optimized further based on testing results until the accuracy of the test output meets the requirements, which results in an SVM pipeline leakage identification model.

The theoretical analysis underlying Equations (23) and (24) indicates that the main factors that affect the SVM model’s performance include the kernel function and the penalty factor *C*. According to the characteristics and ability of the SVM, we took the radial basis kernel as the kernel function in this paper [50,51,52]. The radial basis kernel function is expressed as:

(25) $$K\left(x_{i},x_{j}\right)=\exp\left(-\gamma {∥x_{i}-x_{j}∥}^{2}\right).$$

In this case, the SVM model’s performance is determined by the parameters *C* and γ. To achieve accurate identification results, the optimization process shown in Figure 8 must use the training sample and testing sample to optimize (C,γ). Research has shown that the exponential sequences obtained using *C* and γ can achieve good results. In this paper, we obtained the following parameter values based on the parameter value range: $C=2^{x}\left(x\in \left[-5,15\right]\right)$ and $\gamma =2^{y}\left(y\in \left[-15,5\right]\right)$ [53,54]. Based on the cross-validation grid-search method, we then optimized the SVM parameters. We used the training sample and testing sample to perform testing of an SVM model with different $2^{x}$ and $2^{y}$ combinations and thus obtained the testing accuracy. The final step was to select the *C* and γ values that yielded the optimal cross-validation accuracy, which were selected for identification.

## 5. Simulation Results

### 5.1. Leakage Triggered Networking

In this section, OPNET Modeler14.5 was used to simulate the proposed leakage triggered networking method. The simulation process requires the design and configuration of three different layers. The node layer defines the node behavior and controls the data flow between the different modules in one node. The process layer uses the protocol to perform state conversion for the state machines. The network layer establishes the network topological structure and network layers.

Generally, a ZigBee node model includes an application layer, network layer, MAC layer, and a wireless transceiver. To compile the network power consumption, we added an energy calculation module to the node model. The energy calculation module kept track of the transceiver’s standby, receiving, and transmitting energy consumption via monitoring of the transceiver status. Since the code for the application layer module and network layer module in OPNET was not available, we redesigned the application layer and network layer modules. During the simulation, the coordinator nodes, routing nodes, and terminal nodes had the same node model. The application layer included the source module and sink module. The source module employed the simple_source model, which was a data packet generation module and was responsible for generating data packets with the specified packet size in accordance with the specified packet interval. The sink module was a data packet destruction module and was responsible for destroying data packets that had been transmitted to the destination node, which released internal storage dynamically assigned by the program. The network layer consisted of the network_layer module and mainly served to drive the completion of networking procedures by the MAC module, complete initialization networking and leakage triggered networking, and perform data packet routing in accordance with the AODVjrrouting protocol. The MAC layer employed an 802_15_4_mac module and had a CSMA/CA competitive algorithm. The 802_15_4_mac module performed some networking, multiple access, and sleep management functions via an added sleep state machine. The physical layer employed a wireless_tx/wireless_rx module as a wireless transceiver.

**Figure 9:**
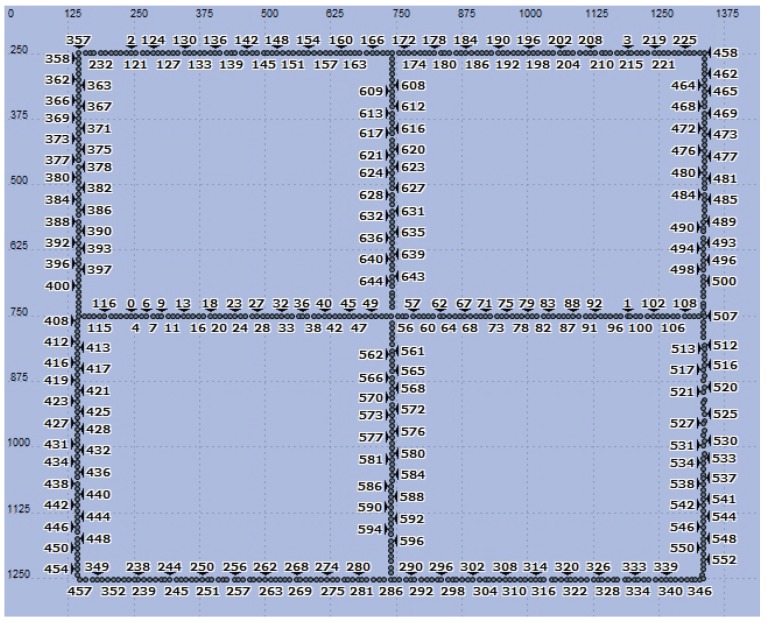
Arrangement of nodes.

The water supply pipeline network shown in Figure 9 was designed for the simulation. The area was 1500 m × 1500 m and contained a total of 644 ZigBee nodes, which included six coordinator nodes and 638 routing and terminal nodes. The coordinator nodes were considered as sink nodes. The distance between adjacent nodes was 10 m; the distance between routing nodes was 50 m; and four terminal nodes were located between each pair of routing nodes. Each of the routing and terminal nodes was also a sensor. The terminal nodes completed the data collection and the detection of the water leakage signal and sent the data to the routing nodes. Then, the routing nodes transmitted the information collected by themselves and the terminal nodes to the sink nodes through multi-hop routing. Finally, the sink nodes sent the information to the background control center to realize the monitoring of the entire network. An RxGroup Configmo module was used to configure the nodes’ single-hop link distance in the simulation scenario, and channel fading employed a free space loss model. The simulation parameters for each layer are shown in Table 4.

**Figure 10:**
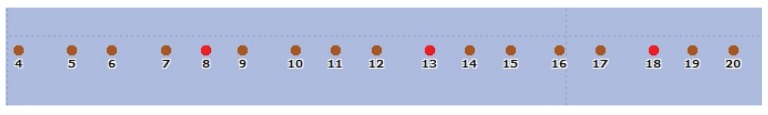
Arrangement of wireless sensor nodes in the vicinity of a leakage point.

The simulation time was 1200 s. First, leakage signal information functions were established in the MAC layer, and a leakage triggered networking experiment was performed via the establishment of leakage point coordinates, signal attenuation coefficients, and the leakage signal detection threshold using the functions. The leakage coordinate was (319, 753), which indicated that the leakage point was located between Nodes 10 and 11, as shown in Figure 10. In the simulation, Node 3 was a coordinator node, Nodes 8, 13, and 18 were routing nodes, and the remaining nodes were terminal nodes. According to the simulation settings, leakage occurred at 700–720 s, and the signal attenuation coefficient and leakage signal detection threshold settings ensured that Routing Nodes 8 and 13 could receive the leakage signal. We also monitored the active and sleep status of the MAC layer to track the nodes’ networking status.

**Figure 11:**
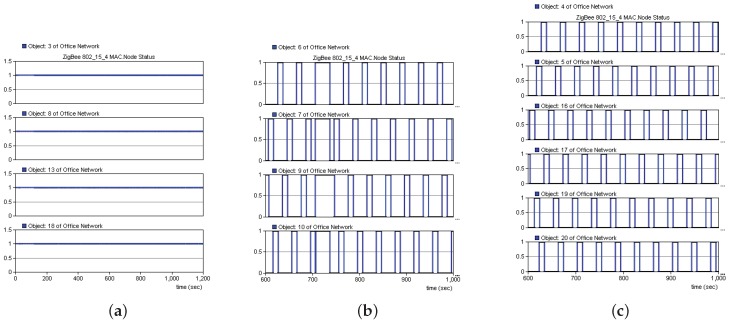
Node status. (**a**) Coordinator and routing nodes. (**b**) Networking nodes. (**c**) Nodes in the normal working state.

Figure 11 shows the working statuses during the 0–1200 s period. The coordinator and routing nodes were consistently in the working state, and the terminal nodes within the routing node network were sequentially working and sleeping. Leakage occurred when the simulation time reached 700 s, and all of the terminal nodes within the network formed by Routing Nodes 8 and 13 entered the working state at that time. Leakage ceased when the simulation time reached 720 s, and the terminal nodes within the network formed by Routing Nodes 8 and 13 resumed the normal working status. The routing nodes on both sides of the leakage point could detect the leakage signal and performed networking when the leakage occurred, whereas the other routing nodes remained in a normal working state. Simulation results indicated that the proposed solution achieved leakage triggered networking by the sensor nodes on both sides of a leakage point, which could further provide data to determine the location of the leakage point.

**Figure 12:**
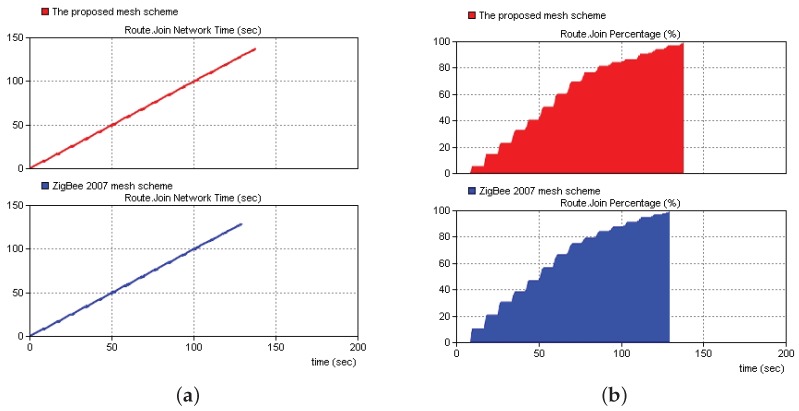
Comparisons of the networking time and proportions of networking nodes for the proposed solution and the ZigBee 2007 solution. (**a**) Networking time. (**b**) Proportion of networking nodes.

Figure 12 shows a comparison of the networking time between the proposed networking solution and the ZigBee 2007 networking solution. The simulation results indicated that the networking time of the proposed solution was slightly greater than that of the ZigBee 2007 solution because of the addition of the RSSI threshold value. However, the networking time of all the nodes increased only by approximately 1.39%. Therefore, the proposed solution could be used in a large scale networking environment.

**Figure 13:**
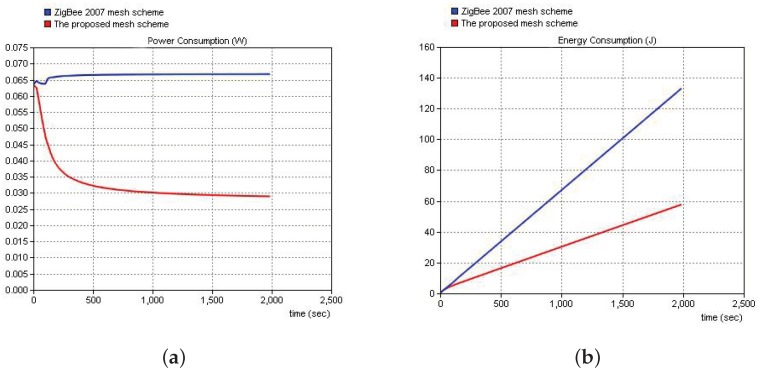
Comparisons of the networking power and energy consumption for the proposed solution and the ZigBee 2007 solution. (**a**) Power. (**b**) Energy.

Figure 13 shows a comparison of the power and energy consumptions of the proposed solution and the ZigBee 2007 solution. Simulation results clearly demonstrated that the proposed solution could reduce the network power and energy consumption and increase the network lifetime through controlling of the polling work of the terminal nodes within their networks.

**Figure 14:**
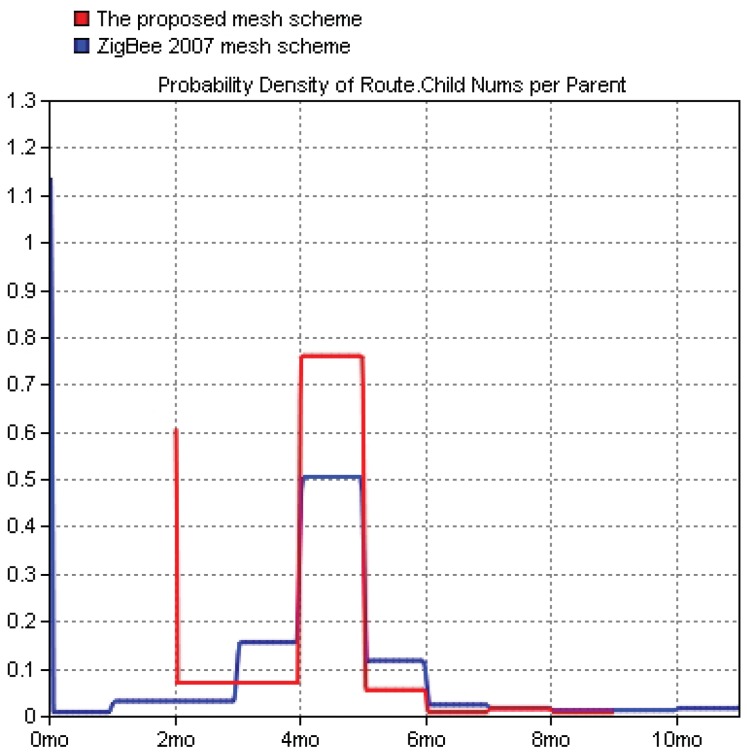
Ratio of terminal nodes carried by routing nodes for the proposed solution and the ZigBee 2007 solution.

Figure 14 shows the percentage of terminal nodes carried by all the routing nodes when the transmission power of the sensor nodes was 1 mW and the RSSI threshold was −68 dBm. In accordance with the channel loss model, the signal transmission distance controlled by the threshold value was approximately 25 m. Because the nodes were spaced at intervals of 10 m in the simulation, four terminal nodes were carried by each routing node. The results shown in Figure 14 indicate that the proposed solution can ensure the number of terminal nodes carried by the routing nodes was more uniform than that in the original solution and could thereby ensure more stable network coverage.

### 5.2. Leakage Identification

The experiment was performed along an exposed aluminum-plastic composite pipe, with a diameter of 27 mm. Moreover, the water-tap was used as the leakage sound source, then the flow rate was adjusted, and the sensor was placed at a distance of 20 cm away from the water-tap. One hundred datasets of leakage signals and non-leakage signals were sampled respectively. Each dataset had a length of 5000, and the data were used to train and optimize the SVM model. In addition, 100 datasets of leakage and non-leakage signals were sampled during the early quiet morning hours, respectively. Therefore, the simulated noise was employed to verify the effectiveness of the proposed leakage detection.

**Figure 15:**
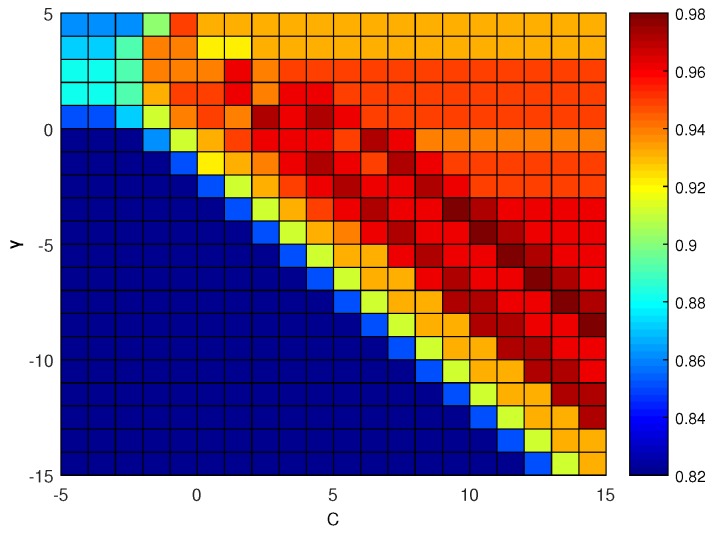
Identification accuracy under different (C,γ) parameter combinations.

At first, 50 datasets were extracted from each of the leakage and non-leakage signals and used to create a training set. The remaining samples were then used to create a testing set. The SVM parameters (C,γ) were set to an integer power of two; the range of *C* was set as $C\in [2^{-5},2^{15}]$; and the range of γ was set as $\gamma \in [2^{-15},2^{5}]$. The grid-search method was used, and 21×21=441 for (C,γ) parameter combinations were used to perform the model training. The detection accuracy is shown in Figure 15. The results indicated that the highest identification accuracy achieved by the proposed algorithm was 98%. In addition, when $C\geq 2^{2}$, $\gamma \leq 2^{0}$, and $2^{1}\leq C\times \gamma \leq 2^{7}$, the SVM model based on the radial basis kernel provided good pipeline leakage signal identification performance.

**Figure 16:**
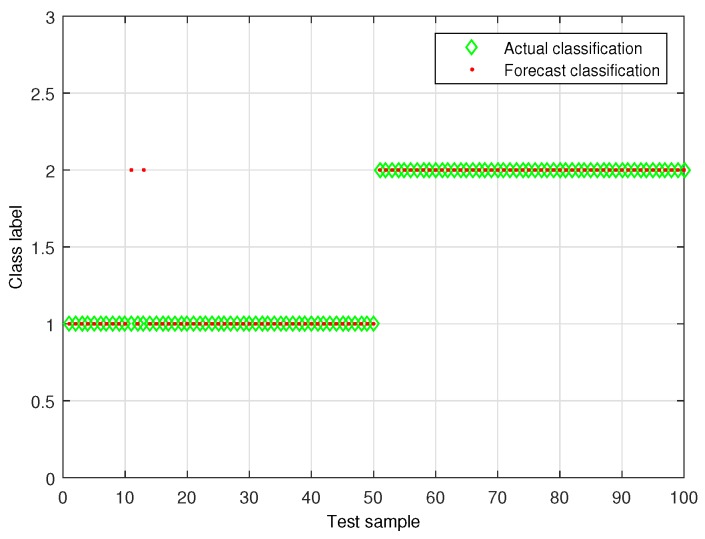
Pipeline leakage and non-leakage detection results.

To verify the effectiveness of the proposed leakage detection, the experiment was performed in which 100 datasets of leakage signals and non-leakage signals were sampled, respectively. Figure 16 shows the detection results for $\left(C,\gamma \right)=\left(2^{9},2^{-4}\right)$ parameter combinations, where the leakage label was 2, and the non-leakage label was 1. The identification results showed that the proposed method only determined two leakage signals to be non-leakage signals and made accurate determinations in all other cases, indicating that the classification accuracy achieved by the proposed algorithm was 98%. Table 5 shows the results obtained by using the proposed algorithm to perform leakage identification after artificial Gaussian noise and impulsive noise were added to leakage signals obtained during a quiet period of time. The results indicated that the proposed water supply pipeline leakage detection method based on the time-frequency features of the signal and SVM could effectively detect pipeline leakage.

## 6. Conclusions

In this paper, an experimental water pipeline leakage detection system based on machine learning and wireless sensors networks was presented. The system employed ZigBee and 4G to acquire and transmitted signals. In addition, a leakage triggered networking method was further proposed to reduce the WSN energy consumption effectively and prolong the system life. To improve the accuracy of water pipeline leakage detection, the proposed system made better use of EMD, ApEn, and PCA of the leak signal and SVM to identify the leakage signal intelligently. Simulation analysis and experimental results indicated that the proposed leakage identification method could effectively identify the water pipeline leakage.

## Figures and Tables

**Table 1:** Join frame.

Field Name	PANID	Des_address	Sou_address	Join_result	Channel
Length	16 bits	16 bits	16 bits	1 bit	32 bits
Instructions	Network ID	Destination	Source	Results	Channel

**Table 2:** Active frame.

Field Name	PAN ID	Sou_address	Act_address
Length	16 bits	16 bits	16 bits
Instructions	Network ID	Source	Active Node

**Table 3:** Wave frame.

Field Name	PAN ID	Des_address	Sou_address	Position	Value
Length	16 bits	16 bits	16 bits	64 bits	128 bits
Instructions	Network ID	Destination	Source	Leakage Point	Information

**Table 4:** Simulation parameter settings.

**Application Layer Parameters**	
Packet Size	Constant (512)
Packet Interarrival Time	Exponential (5)
Start Time	120 s
Stop Time	Infinity
**MAC Layer Parameters**	
ACK Wait Duration	0.05 s
Minimum Backoff Exponent	3
Maximum Number of Backoffs	4
Channel Sensing Duration	0.5
**Physical Layer Parameters**	
Transmission Bands	2450 MHz Band
Data Rate	240 kbps
Packet Reception-Power Threshold	−85 dBm
Transmission Power	1 mW
Receive Power	0.4 mW
Idle Power	0.1 mW

**Table 5:** Leakage signal identification rate in an environment containing Gaussian noise and impulse noise.

		SNR(dB)	−12	−9	−6	−3	0	3	6	9	12
	(c,g)										
	$\left(2^{9},2^{-4}\right)$		34	42	60	83	85	88	91	93	96
	$\left(2^{10},2^{-5}\right)$		34	42	60	82	85	87	91	92	96
	$\left(2^{11},2^{-6}\right)$		34	42	60	82	85	87	89	93	96
Gaussian Noise	$\left(2^{12},2^{-7}\right)$		37	42	60	82	84	87	88	93	95
	$\left(2^{13},2^{-8}\right)$		34	42	60	82	85	87	91	93	96
	$\left(2^{14},2^{-9}\right)$		34	42	59	82	85	87	91	93	96
	$\left(2^{15},2^{-10}\right)$		34	42	59	82	85	87	91	83	96
	$\left(2^{9},2^{-4}\right)$		31	32	56	84	85	88	90	94	96
	$\left(2^{10},2^{-5}\right)$		31	32	54	82	84	87	89	93	95
	$\left(2^{11},2^{-6}\right)$		31	32	59	81	84	87	90	93	95
Impulsive Noise	$\left(2^{12},2^{-7}\right)$		31	32	56	81	85	87	90	93	95
	$\left(2^{13},2^{-8}\right)$		31	32	55	81	84	87	90	93	95
	$\left(2^{14},2^{-9}\right)$		31	33	55	81	84	87	91	93	96
	$\left(2^{15},2^{-10}\right)$		31	32	55	80	84	87	91	93	95

## Abbreviations

The following abbreviations are used in this manuscript:

WSNs	Wireless sensor networks
SVM	Support vector machine
LPCC	Linear predictive coding coefficient
HMM	Hidden Markov model
PCA	Principal component analysis
LOS	Line-of-sight
NLOS	Non-line-of-sight
RSSI	Received signal strength indicator
EMD	Empirical mode decomposition
IMFs	Intrinsic mode functions
ApEn	Approximate entropy
SD	Standard deviation
